# Modified conditioning regimen with chidamide and high‐dose rituximab for triple‐hit lymphoma

**DOI:** 10.1111/jcmm.16999

**Published:** 2021-10-25

**Authors:** Junnan Kang, Yizhuo Zhang, Sa Ding, Kalbinur Yasheng, Yueyang Li, Yong Yu, Yafei Wang, Chen Tian

**Affiliations:** ^1^ Department of Hematology Tianjin Medical University Cancer Institute and Hospital National Clinical Research Center for Cancer Key Laboratory of Cancer Prevention and Therapy Tianjin China; ^2^ Department of Oncology Hetian District People's Hospital Hotan China

**Keywords:** chidamide, conditioning regimen, high dose rituximab, triple‐hit lymphoma

## Abstract

Triple‐hit lymphoma (THL), which is classified into high‐grade B‐cell lymphoma with rearrangements of MYC, BCL2 and BCL6, presents aggressive biological behaviour. High‐dose chemotherapy followed by autologous hematopoietic stem cell transplantation (auto‐HSCT) is considered to be one of the recommended treatment options. Here, we reported 3 THL patients received carmustine, etoposide, cytarabine and cyclophosphamide (BEAC) combined with chidamide and high‐dose rituximab conditioning regimen and found that this conditioning showed good efficacy and tolerance without increase of adverse events.

Triple‐hit lymphoma (THL) is considered as a distinct entity, which presents a poor prognosis after standard chemoimmunotherapy, accounting for approximately 5–10% of diffuse large B‐cell lymphoma (DLBCL). In 2016, the revised World Health Organization (WHO) guidelines classified this special type to a new category named high‐grade B‐cell lymphoma with rearrangements of MYC, BCL2 and BCL6. Currently, there is no standard treatment for THL, while high‐dose chemotherapy followed by autologous hematopoietic stem cell transplantation (auto‐HSCT) is considered to be one of the recommended treatment options. Although BEAM (BCNU, etoposide, cytarabine and melphalan), BEAC (BCNU, etoposide, cytarabine and cyclophosphamide) and CBV (cyclophosphamide, BCNU and etoposide) are considered to be classical conditioning regimens for lymphoma, novel and improved conditioning regimens to achieve better prognosis are carried out in many transplantation centres. In order to improve the prognosis of THL, we innovatively added chidamide, a histone deacetylase (HDAC) inhibitor, combined with high‐dose rituximab (500 mg/m^2^) to BEAC regimen. Here, we reported 3 THL cases received standard chemotherapy and then BEAC conditioning regimen combined with chidamide and high‐dose rituximab followed by auto‐HSCT.

## 1 CASE 1

**FIGURE 1:**
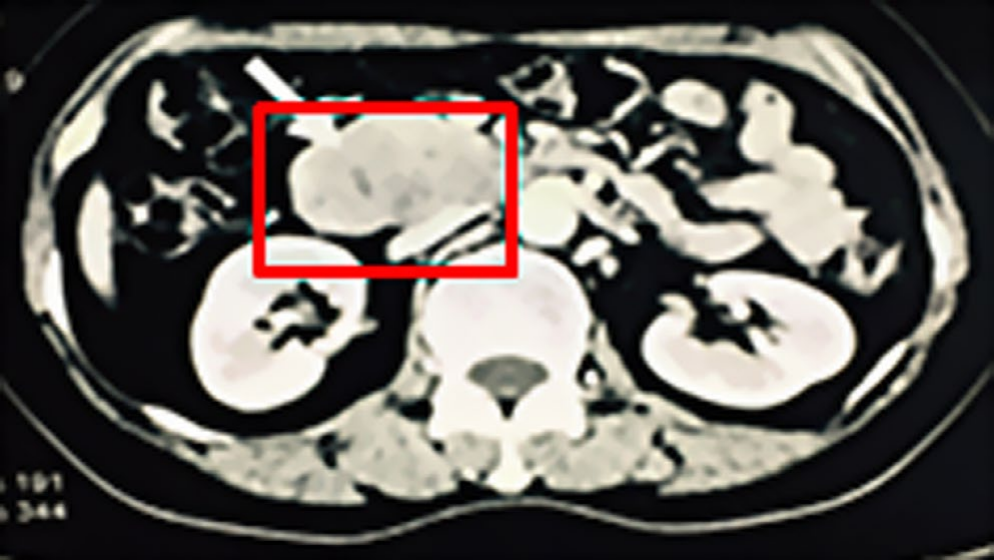
CT scan showed a mass in ampullary and head of pancreas

A 56‐year‐old female presented with loss of appetite, icterus and clay stool in July 2015. Abdominal enhanced computed tomography (CT) scan suggested that the ampullary and head of pancreas presented irregular low‐density shadow, considering ampullary carcinoma (Figure 1). Lactate dehydrogenase (LDH) level was 4500 U/L (normal 140–270 U/L), and serum ferritin (FER) level was 550 µg/L (normal 12–150 µg/L), while her other laboratory results were normal. She received pancreaticoduodenectomy, and the biopsy of the duodenal mass revealed diffuse large B‐cell lymphoma (DLBCL). Lymphoma cells were positive for CD20, CD10, Bcl‐2, Bcl‐6, cyclinD1, Ki67, c‐MYC and negative for CD30, CD5 and MUM‐1. Fluorescence in situ hybridization (FISH) showed Bcl‐2, Bcl‐6 and c‐MYC rearrangements. Bone marrow aspiration and trephine biopsy showed no infiltration. Post‐operative ^18F^‐fluorodeoxyglucose (FDG) positron emission tomography (PET) scanning showed increased FDG uptake in the residual pancreas, small curvature of stomach and retroperitoneal. A diagnosis of DLBCL, germinal centre B‐cell (GCB) subtype, triple‐hit lymphoma was made. After four cycles of R‐CHOP, she achieved partial remission (PR). However, B symptoms of fever, night sweats and weight loss were obviously aggravated. So two cycles of R‐DAEPOCH regimen were given to her and PET/CT results showed complete remission (CR).

Modified BEAC conditioning regimen added with chidamide 10 mg/d, −14d~−2 d and rituximab 500 mg/m^2^, −1d, +8d was given to her followed with auto‐HSCT. The time to neutrophil and platelets engraftment showed no extension. No severe adverse events (AEs) were observed. She is still in CR state till now without relapse. The duration of remission time for her is over 5 years.

## 2 CASE 2

A 58‐year‐old female was admitted to our hospital with low‐grade fever and fatigue. Ultrasound showed multiple lymphadenopathy in her neck, armpit, groin, iliac fossa and retroperitoneum. Laboratory tests including blood routine test and LDH were normal. The biopsy of lymphonode on the neck was positive for CD20, CD79a, PAX‐5, Ki67 (90%+), Bcl‐2, Bcl‐6, CD10, c‐MYC and negative for CD3, CD5, CyclinD1, CD23, CD21, MUM‐1, suggesting DLBCL, GCB subtype. FISH showed Bcl‐2, Bcl‐6 and c‐MYC rearrangements. A diagnosis of DLBCL, GCB subtype, triple‐hit lymphoma was made. Then, she received six cycles of R‐DAEPOCH and achieved CR.

Modified BEAC conditioning regimen with chidamide 10 mg/d, −15d~−3d and rituximab 500 mg/m^2^, −1d, +8d was given to her followed by auto‐HSCT. Neutrophil and platelet engraftment restored on +14d and +18d. There was no complication in the aplastic period except neutropenic fever. She is still in CR state till now without relapse. The duration of remission time for her is four and a half years.

## 3 CASE 3

**FIGURE 2:**
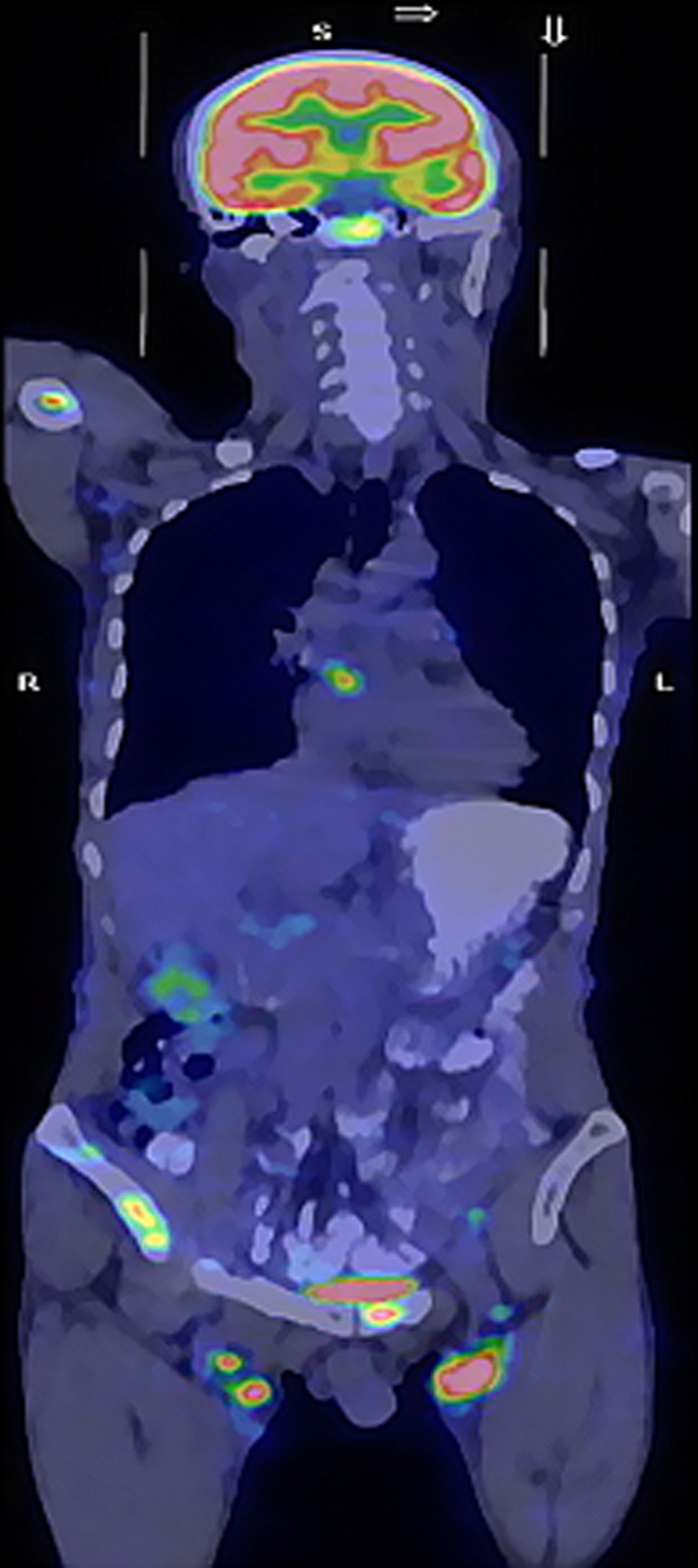
PET/CT showed multiple subcutaneous masses all over the body

A 56‐year‐old male with multiple flat masses in his skin of right limb, chest, abdomen and back was administered in our hospital in November 2015. On examination, he had painless lymphadenopathy in both groin. PET/CT indicated systemic high‐density subcutaneous nodules with increased FDG uptake (Figure 2). LDH level was 1124 U/L and FER level was 480 µg/L. Bone marrow examination as well as cranial magnetic resonance imaging (MRI) showed no abnormalities. The skin mass biopsy revealed DLBCL, positive for CD20, Ki67 (60–70%+), CD10, Bcl‐2, Bcl‐6, MYC and negative for CD3, CD56, CD34, CD123, CD21, MUM‐1, TdT and CD5. FISH showed Bcl‐2, Bcl‐6 and c‐MYC rearrangements. So he was diagnosed as DLBCL, GCB subtype and triple‐hit lymphoma.

He was treated with four cycles of R‐DAEPOCH but his disease was progressed. Then he received 2 cycles of R‐MA (methotrexate, cytarabine) combined with chidamide of 20 mg twice a week and he received PR. BEAC conditioning regimen added with chidamide and rituximab were given to him followed with auto‐HSCT. However, the patient developed a rash on the neck and upper arm when his hematopoietic reconstruction was not restored indicating disease progression. Two months later, although his hematopoietic reconstruction restored after HSCT, the rash spread his whole body and he had high fever not considering infection, resulting in increasingly weak. He died owing to disease progression.

Double hit lymphoma (DHL)/THL refer to B‐cell lymphoma with MYC accompanied with Bcl‐2 and/or Bcl‐6 gene rearrangements.^1^ THL patients often have advanced stage and poor prognosis, and there is no adequate consensus therapy to treat them.^2^ A systematic meta‐analysis revealed that first‐line treatment with R‐EPOCH significantly reduced the risk of progression compared with R‐CHOP; however, OS was not significantly different across treatment approaches.^3^ It was reported that DHL/THL patients who did not achieve CR had significantly prolonged overall survival (OS) after HSCT consolidation therapy, suggesting that THL patients may benefit from HSCT.^4^ Landsburg et al revealed that consolidative auto‐HSCT was not associated with improved 3‐year OS.^5^ A phase III randomized study (SWOG S9704) found that there was a trend favouring outcomes after consolidative auto‐HSCT in double protein‐expressing lymphoma (DPL) and MYC protein overexpressing patients, whereas all DHL patients have died irrespective of HSCT.^6^ A Japanese study compared auto‐HSCT and allogeneic hematopoietic stem cell transplantation (allo‐SCT) in DHL patients and found that auto‐HSCT may be effective for chemosensitive and relapsed or refractory DHL patients. Since most patients received allo‐HSCT not in CR, the outcome of allo‐HSCT was unsatisfactory due to high non‐relapse mortality (NRM) and early relapse.^7^

It is reported that HDAC inhibitors can overcome congenital and acquired rituximab resistance in patients with B‐cell lymphoma and augment the cytotoxic activity of rituximab by upregulating the expression of CD20 in lymphoma cells. Chidamide, a new HDAC inhibitor, could be a potent therapeutic agent to treat DLBCL by inducing the DLBCL cell apoptosis by inhibiting the HDACs/STAT3/Bcl‑2 pathway.^8^ Hu et al reported that chidamide combined with lenalidomide could achieved excellent outcomes in heavily pretreated aggressive DLBCL patients.^9^

Rituximab revolutionized the treatment landscape of B‐NHL. However, whether B‐NHL patients could benefit from rituximab‐based conditioning regimen are still unknown. Jagadeesh et al reported that the addition of high‐dose rituximab (>375 mg/m^2^) to the BEAM conditioning regimen had no impact on transplantation outcomes.^10^ However, Shi et al demonstrated that high‐dose rituximab followed with auto‐HSCT is a feasible and promising treatment for relapsed or refractory DLBCL.^11^ Chen et al reported that primary central nervous system (CNS) DLBCL patients gained perfect efficacy receiving high‐dose rituximab (1000 mg/m^2^) followed with auto‐HSCT.^12^ Laport et al revealed that high‐dose rituximab conditioning conferred high CR rates, low relapse/progression incidence and excellent survival probabilities in heavily pretreated patients.^13^

BEAC was one of the most commonly used conditioning regimens before auto‐HSCT, given its optimal efficacy and tolerability. However, more tolerated and efficacious modified conditioning regimens aiming to obtain a higher anti‐lymphoma activity are still indispensable, especially for DHL/THL. In our study, 2 patients showed good response to chemotherapy and achieved CR before auto‐HSCT. BEAC combined with chidamide and high‐dose rituximab conditioning regimen did not extend the time to completed haematopoietic engraftment. No other significant extrahaematological toxicities emerged, suggesting good tolerance. Both of them are still in CR state. However, the third patient died after auto‐HSCT, suggesting that patients who were insensitive to chemotherapy had poor prognosis.

## CONFLICT OF INTEREST

The authors declare that they have no conflicts of interest.

## AUTHOR CONTRIBUTIONS

**Junnan Kang:** Formal analysis (lead); Writing‐original draft (lead). **Yizhuo Zhang:** Investigation (equal); Supervision (equal); Validation (equal). **Sa Ding:** Writing‐original draft (equal). **Kalbinur Yasheng:** Writing‐original draft (equal). **Yueyang Li:** Writing‐original draft (equal). **Yong Yu:** Writing‐original draft (equal). **Yafei Wang:** Writing‐review & editing (equal). **Chen Tian:** Funding acquisition (lead); Supervision (lead); Validation (lead); Writing‐review & editing (lead).

## CONSENT FOR PUBLICATION

Not applicable.

## ETHICS APPROVAL AND CONSENT TO PARTICIPATE

This study was subject to approval by the Research Ethics Committee of Tianjin Medical University Cancer Institute and Hospital.

## Data Availability

The data sets used and/or analysed during the current study are available from the corresponding author on reasonable request.
